# Nutrition and degeneration of articular cartilage

**DOI:** 10.1007/s00167-012-1977-7

**Published:** 2012-04-04

**Authors:** Yuze Wang, Lei Wei, Lingyuan Zeng, Dongdong He, Xiaochun Wei

**Affiliations:** 1 Department of Orthopaedics, The Second Hospital of Shanxi Medical University, 382 Wuyi Road, Taiyuan, 030001 Shanxi People’s Republic of China; 2 Department of Orthopaedics, The Warren Alpert Medical School of Brown University/Rhode Island Hospital, Suite 402A, 1 Hoppin Street, Providence, RI 02903 USA; 3 Shanxi Key Lab of Bone and Soft Tissue Injury Repair, 382 Wuyi Road, Taiyuan, 030001 Shanxi People’s Republic of China

**Keywords:** Articular cartilage, Nutrition source, Cartilage lesion

## Abstract

**Purpose:**

To determine the importance of synovial fluid (SF) or subchondral bone marrow (BM) as nutrition sources in cartilage degeneration.

**Methods:**

Ninety-five-month-old male rabbits were randomly divided into 5 groups according to sources of nutrition: SFBM-both; BM-only; SF-only; None-SFBM; and Free plug (unrestricted). Nutrition to 4-mm-diameter cylindrical osteochondral plugs created on the trochlea of the distal femurs was obstructed by Polyvinyl Chloride (PVC) cap. Cartilage changes were assessed after 4, 8, and 12 weeks by histology, immunohistochemistry, and real-time PCR.

**Results:**

Cartilage in the BM-only group suffered the greatest damage, followed by the None-SFBM and SF-only groups. Apoptosis was increased in the BM-only and None-SFBM groups compared with others. Cartilage was significantly thinner at all time points in the BM-only and None-SFBM groups when compared with SFBM-both and Free plug, whereas in the SF-only group, this difference occurred after 8 weeks. Compared with SFBM-both and Free plug, expression of collagen II and aggrecan mRNAs in all groups was decreased but MMP-3 increased, respectively.

**Conclusion:**

Our data indicate that SF-derived nutrition is the dominant source of sustenance for adult cartilage structure and function. Cartilage damage is observed when the only nutrition source is the BM.

**Electronic supplementary material:**

The online version of this article (doi:10.1007/s00167-012-1977-7) contains supplementary material, which is available to authorized users.

## Introduction

Articular cartilage is an avascular tissue [15] nourished by two potential pathways: diffusion from subchondral bone vessels and diffusion from the synovial fluid. The relative importance of these pathways is controversial [12]. Collected evidence indicated that deficiencies in the nutrition of cartilage could be one of the chief reasons for this tissue’s degeneration [10, 14, 15, 25]. Autoradiographic and tracer studies in animals have indicated that while immature articular cartilage can be nourished via both synovial and subchondral routes, articular cartilage in mature animals derives its nutrition exclusively from synovial fluid, because of the calcified barrier with the subchondral division [21–23]. Apart from these, human studies by Maroudas and Bullough [20] provided evidence indicating that it was only in immature human specimens that soluble molecular substances could penetrate from the marrow cavity into cartilage. However, Greenwald and Haynes [9] employed non-toxic fluorescent and tracer technology to visualize blood movement in the human femoral head and observed that the fluorescent substances within the bone marrow could penetrate into cartilage tissues in adult humans [1, 2, 9].

While these studies suggest that articular cartilage can be nourished by both subchondral bone marrow and synovial routes, the relative importance of these two routes remains uncertain. Our hypothesis is that both the routes of nutrition from the subchondral bone marrow and the synovial fluid play a critical role in maintaining adult normal cartilage homeostasis and function. The aim of this study was to determine the relationship between the nutritional pathways and the degeneration of articular cartilage. To determine which of the routes of nutrition (subchondral bone marrow or synovial fluid) plays a critical role in maintaining adult normal cartilage homeostasis and function, we deprived the nutrition of articular cartilage by SF-only, BM-only, or both-SFBM, using a PVC cap. The effects of nutrition deprivation were evaluated at different time points by gross tomography, histological analyses, immunohistochemistry, and real-time PCR.

## Materials and methods

### Animal model

**Fig. 1:**
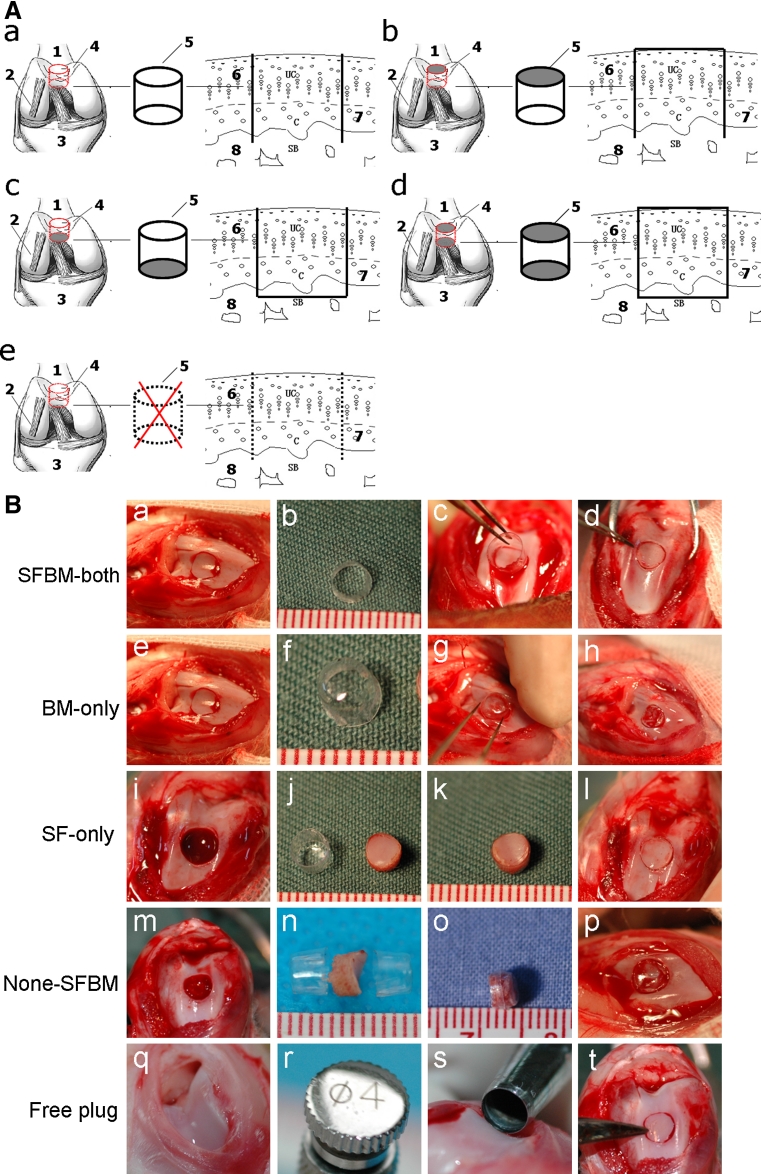
**A** Diagram of animal model. **a** SFBM-both group (maintaining nutrition from both SF and BM with the PVC tube open at both ends); **b** BM-only group (nutrition from BM-only, with the PVC tube blocked at the synovial fluid end); **c** SF-only group (nutrition from SF-only, with the PVC tube blocked at the bone marrow end); **d** None-SFBM group (obstructed nutrition from both SF and BM by blocking both ends of the PVC tube); **e** Free plug group (nutritional access unrestricted by replacing the osteochondral plug without surrounding it in a PVC tube); *1*. Femur. *2*. Lateral collateral ligament. *3*. Tibia. *4*. Femoral trochlea. *5*. PVC cup or tube. *6*. Superficial cartilage. *7*. Calcified layer. *8*. Subchondral bone. **B** Animal model surgeries

**Fig. 2:**
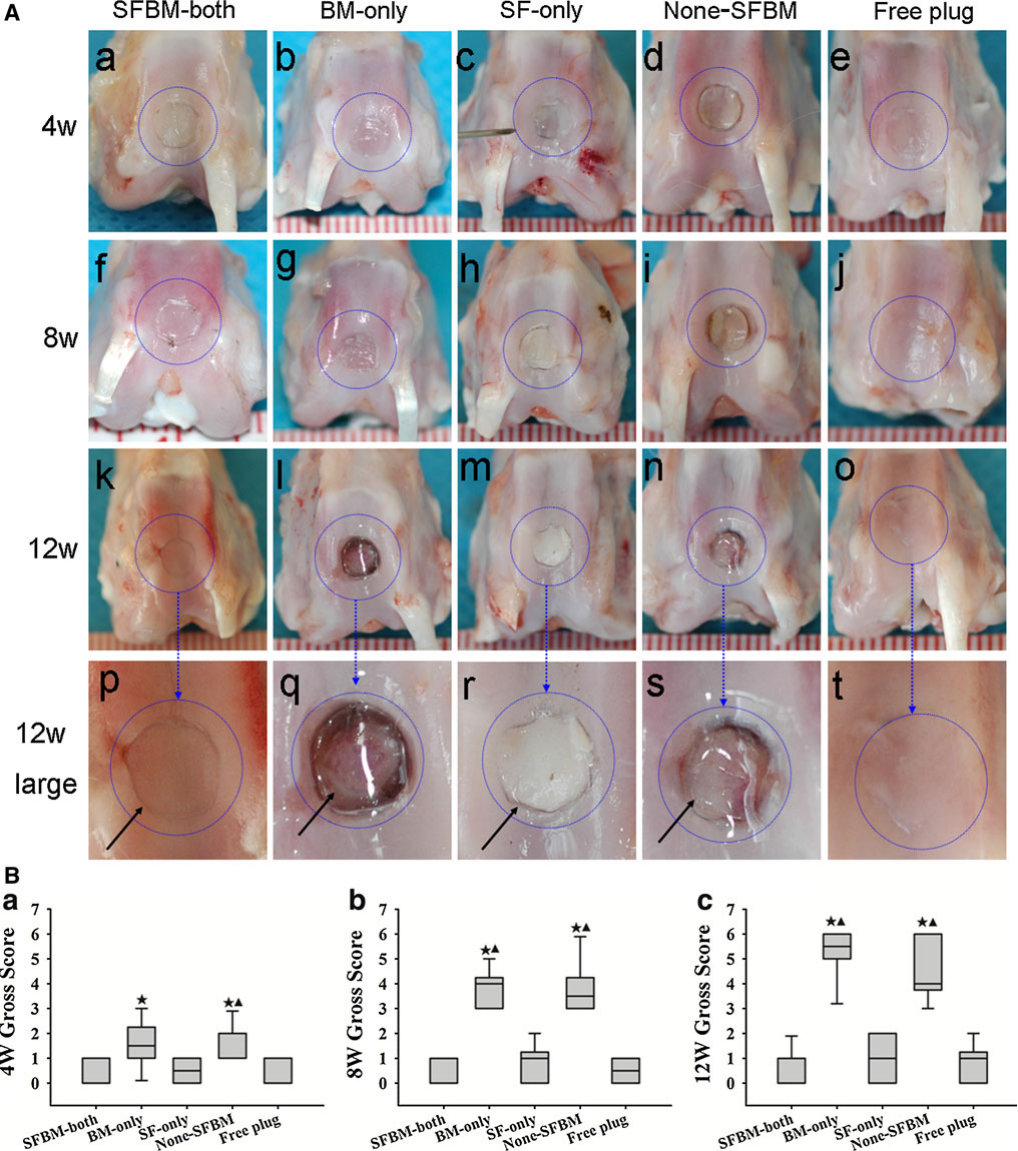
**A** Macroscopic appearance. **B** Gross Score showed a marked increase in cartilage damage in the BM-only and None-SFBM groups at 4, 8, and 12 weeks after the operation. Medians ± Interquartile range (M ± QR)* star* *P* < 0.005, vs. SFBM-both group;* triangle* *P* < 0.005, vs. SF-only group

Ninety-five-month-old healthy male New Zealand white rabbits (2.8 ± 0.3 kg) were obtained from the Animal Research Center of Shanxi Medical University. All procedures were approved by the Animal Care and Use Committee of Shanxi Medical University. A 3-mm parapatellar incision was made to expose the knee joint after anesthesia. Using a precision 4.0-mm trephine (Cat. #530-745-06, Six–Six Visual Machinery Company, JianShu, China, wall thickness 0.1-mm), 3.8-mm-diameter × 4.0-mm-long osteochondral plugs were removed from the trochlea of distal femurs just above the lateral ligament (Fig. 1A). The plugs were replaced in their original anatomic orientation, but with nutritional access restricted with interposed PVC inserts. Group allocation was random. In the SFBM-both group (*n* = 18), where nutritional access was maintained at the synovial and bone marrow surfaces, the osteochondral bone plug was replaced after insertion into a length of PVC tube (0.1 mm wall thickness, 4 mm diameter and 4 mm depth, Shanxi Pharmaceutical company, Taiyuan, China) open at both ends (Fig. 1A-a, B-a–d). In the BM-only (*n* = 18) and SF-only (*n* = 18) groups, nutritional access was allowed only at the bone marrow and synovial surfaces, respectively, as the osteochondral bone plugs were replaced after insertion into PVC cylinders capped at the synovial and bone marrow ends, respectively (Figs. 1A-b, B-e–h, A-c, 2B-i–l, respectively). In the None-SFBM group (*n* = 18), nutritional access was completely restricted by replacing the plugs after insertion into PVC cylinders capped at both the synovial and bone marrow ends (Fig. 1A-d, B-m–p). Finally, in the Free plug group, nutritional access was unrestricted, as the osteochondral plug was reinserted directly (Fig. 1A-e, B-q–t). The knee joints were collected at 4, 8, and 12 weeks post-operatively.

### Gross observation

The gross appearance of the cartilage was scored with a gross grading system as published before [4]. Grading of the gross appearance of the cartilage was performed by direct observation and by studying detailed photographs of each specimen. The gross appearance scoring was performed as follows: normal/glistening cartilage, 0; pale, as compared with the surrounding cartilage, 1; yellowish, 2; pitted, 3; ulcerated, 4; subchondral bone visible, 5; cartilage absent, 6. The maximum (worst) score is 6. Three independent and blinded observers scored each joint, and the scores for the three observers were averaged within each joint.

### Histological analysis

The specimens were fixed, decalcified, and embedded in paraffin. The 6-μm sections were stained with Safranin O/fast green, H&E, and then scored by Mankin grading system [8, 16, 18, 19]. The grading system is based on the assessment of the structure (normal, 0; surface irregularities, 1; pannus and irregularities, 2; clefts to transitional zone, 3; clefts to radial zone, 4; clefts to calcified zone, 5; complete disorganization, 6), cells (normal, 0; hypercellularity, 1; cloning, 2; hypocellularity, 3), histochemistry (normal, 0; slight reduction in staining, 1; moderate reduction, 2; severe reduction, 3; no staining, 4), and tidemark integrity (normal, 0; crossed by vessels or disrupted, 1). The maximum (worst) score is 14. The thickness measurements were made according to a previously described method [11]. Cartilage thickness was measured at three sections per sample, chosen randomly, where the cartilage length was more than 3 mm, by Imaga-Pro6.3 Software with microscopy (Olympus BX51; Olympus, Tokyo, Japan). The cartilage thickness was measured from the cartilage surface to the tide line on cartilage sagittal slices. (Suppl. Fig. 3). The thickness of the three sections was averaged within each joint. Two independent and blinded observers scored each section, and the scores from the two observers were averaged within each joint. There is no statistic difference between the test–retest and the two observers measurement determined by Rank correlation test (spearman rank correlation coefficient >0.82) and Bland–Altman plot analysis (within or less the range of ±1.96 standard deviation), respectively.

### Immuno-histochemistry

Only 4-week samples were used for type II collagen staining (Histostain-SP Kits, Zymed, Carlsbad, CA Cat. #95–9943) and real-time PCR, due to 8- and 12-week cartilage samples being too damaged for these analyses, especially in the BM-only group. The sections were incubated with a monoclonal mouse Ab against Collagen Type II(20μg/mL; Cat. #cp18 Calbiochem, Japan), at 4 °C overnight. The negative control sections were incubated with isotype control (25μg/mL; Cat. #MAB002 R&D Systems, Inc., Minneapolis, MN) in 0.01 M PBS. Thereafter, the sections were treated sequentially with ready-to-use biotinylated secondary antibody and streptavidin-peroxidaseconjugate, followed by standardized development in DAB. Photography was performed with Olympus BX51.

### TUNEL assay

Apoptosis was examined using Apop Tag Peroxidase In Situ Detection kit according to the manufacturer’s instructions (Cat. #11684817910; Roche Diagnostics, Basel, Switzerland). Sections were assessed by two blinded observers using an Olympus BX51 microscope at 400× magnification. The percentage of positively stained chondrocytes was calculated by counting the number of positively and negatively stained cells in each slide.

### Real-time PCR

Frozen cartilage (0.5 g) samples were crushed to a powder using a mortar and pestle (*n* = 3, 6 joints from both sides). Total RNA was isolated using Trizol reagent (15596–026, INVITROGEN, Carlsbad, Carlsbad, USA). One microgram total of RNA was reverse-transcribed with the iScript^™^ cDNA Synthesis Kit (K1642, FERMENTAS, MARYLAND, USA). Real-time quantitative PCR amplification was performed using the QuantiTect SYBR Green PCR kit (K0251, FERMENTAS, MARYLAND, USA). mRNA levels were normalized to GAPDH and calculation of mRNA values was performed as previously described [29–32]. The cycle threshold (Ct) values for GAPDH and that of samples were measured and calculated using computer software (IQ50, Bio-Rad, USA). Relative transcription levels were calculated as *x* = 2^−ΔΔCt^, in which ΔΔCt = ΔE−ΔC, and ΔE = Ctexp−CtGAPDH; ΔC = Ctctl−CtG. The primer sequences used for RT-PCR were as follows: Col-2 forward 5′-ACACTGCCAACGTCCAGATG-3′ and reverse 5′-GTGAT GTTCTGGGAGCCCTC-3′ (D83228); AGG forward 5′-TCTACCGCTGTGAGGTGAT GC-3′ and reverse 5′-TTCACCACGACCTCCAAGG-3′ (L38480); MMP-13 forward 5′-ACACCGGATCTGCCAAGAGA-3′ and reverse 5′-CTGGAGAACGTGATTGGAGT CA-3′ (001082037); GAPDH forward 5′-GGTGAAGGTCGGAGTGAACG-3′ and reverse 5′-AGTTAAAAGCAGCCCTGGTGA-3′(L23961).

### Statistical analysis

The data of histologic scores are expressed as medians ± interquartile range and analyzed by the nonparametric Kruskal–Wallis test, followed by Bonferroni post hoc test. Other data are displayed as means ± standard deviations and analyzed by one-way analysis of variance (ANOVA) post hoc test of Tukey’s method. The statistical significance level of nonparametric Kruskal–Wallis test and one-way ANOVA was set at *P* < 0.005 and *P* < 0.05, respectively.

## Results

### Gross observation and score

A pilot study demonstrated the definite efficacy of the PVC cap at deprivation of nutrition (Supplement 2). In this study, we found that the gross score of BM-only was increased from 4 weeks (1.5 ± 1.3) to 12 weeks (5.5 ± 1.0) and the gross score of None-SFBM was increased from 4 weeks (2.0 ± 1.0) to 12 weeks (4.0 ± 2.3), respectively. And at 12 weeks, the gross histological grading score revealed that the greatest lesions were in the BM-only joints (5.5 ± 1.0, *P* < 0.01), followed by moderate lesions in the None-SFBM joints (4.0 ± 2.3, *P* < 0.01), and minor damage in the SF-only joints (1.0 ± 2.0, n.s), in comparison with the SFBM-both and Free plug joints (Fig. 2A, B). At 12 weeks, the gross score of BM-only (*P* < 0.01) and None-SFBM (*P* < 0.01) groups was increased compared with the SF-only group (Table 1).

**Table 1:** Different group Gross Score in 4, 8, and 12 weeks after the operation (M ± QR)

Group (weeks)	SFBM-both	BM-only	SF-only	None-SFBM	Free plug
4	0.0 ± 1.0	1.5 ± 1.3*	0.5 ± 1.0	2.0 ± 1.0*^,▲^	0.0 ± 1.0
8	0.0 ± 1.0	4.0 ± 1.3*^,▲^	1.0 ± 1.3	3.5 ± 1.3*^,▲^	0.5 ± 1.0
12	1.0 ± 1.0	5.5 ± 1.0*^,▲^	1.0 ± 2.0	4.0 ± 2.3*^,▲^	1.0 ± 1.3

* *P* < 0.005, vs. SFBM-both group; ^▲^ *P* < 0.005, vs. SF-only group

### Histological results

**Fig. 3:**
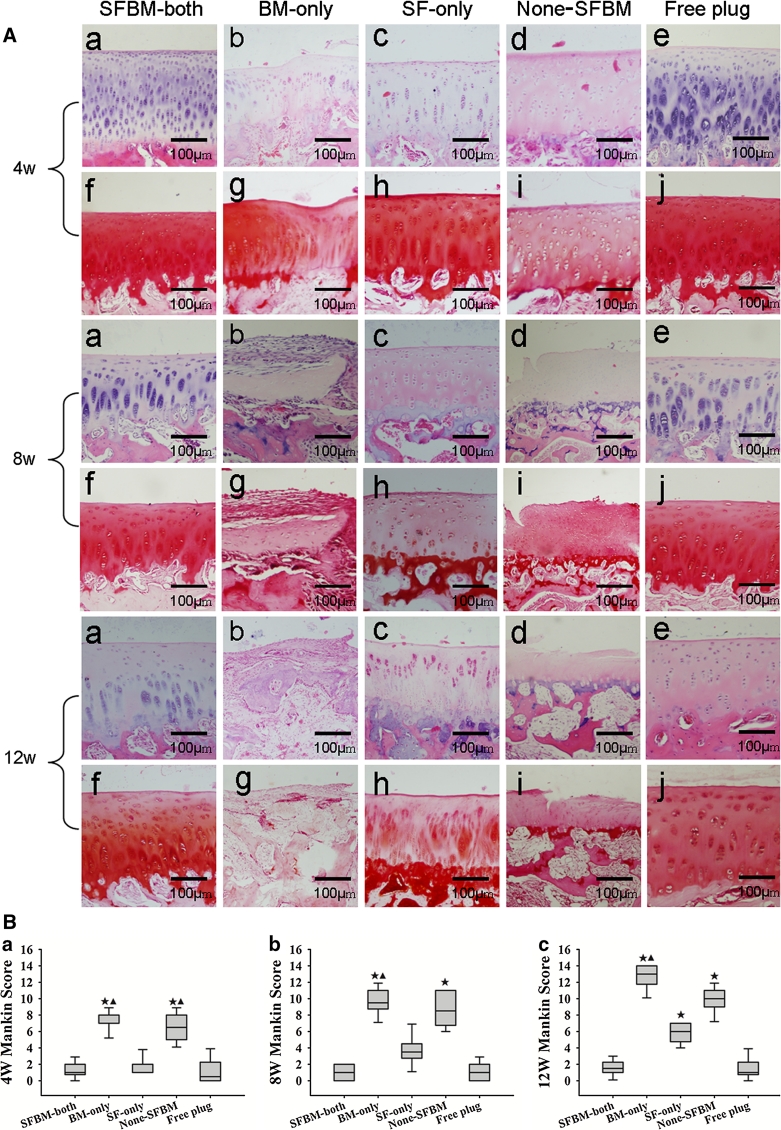

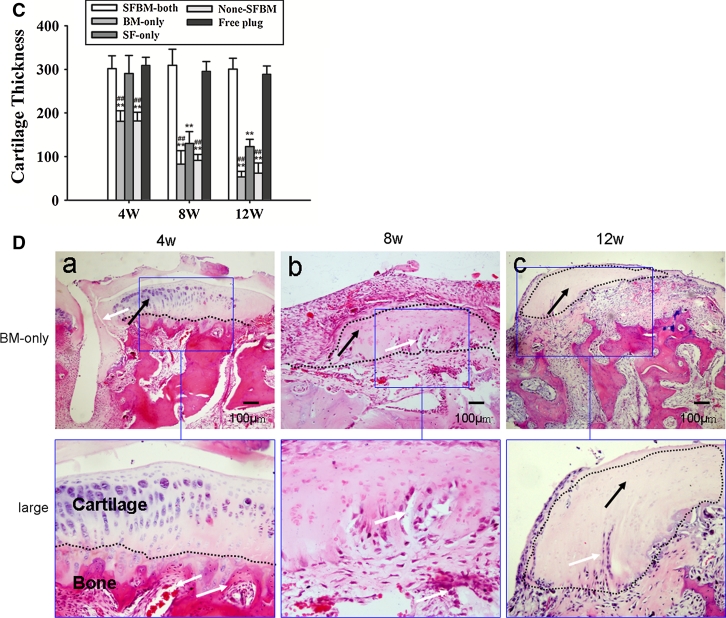
**A** H&E and Safranin O staining showed a marked increase in cartilage damage for BM-only, None-SFBM, and SF-only groups at 4, 8, and 12 weeks. **B** Mankin score showed a marked increase in cartilage damage for BM-only and None-SFBM at all time points, but the difference was only found in the SF-only group at week 12 compared with the SFBM-both and Free plug groups. *Star*  *P* < 0.005, vs. SFBM-both group;* triangle*  *P* < 0.005, vs. SF-only group. **C** Deprivation of nutrition resulted in decreased cartilage thickness. * *P* < 0.05 vs. SFBM-both group, ** *P* < 0.01, vs. control group; # *P* < 0.05 vs. SF-only group, ## *P* < 0.01, vs. SF-only group. **D** Blood vessels, BM and channels at the osteochondral junction from the BM-only group at 4, 8, and 12 weeks after operation. A *dotted line* (*tide line*) separates the underlying subchondral bone and calcified cartilage from the uncalcified cartilage. **a** The bone marrow starts to invade calcified cartilage and subchondral bone. **b** Blood vessels crossing into or approaching the residual cartilage (*white arrow*). Calcified cartilage disappears and there is loss of full thickness cartilage (*black arrow*). Residual cartilage was surrounded by fibril tissue (*black dotted circle*). **c** Cartilage was replaced by fibril tissue and BM

H&E and Safranin O staining showed that cartilage architecture gradually degenerated with time under deprived nutrition conditions (Fig. 3A). As graded by the Mankin score, the greatest cartilage lesions were found in both the BM-only (14.0 ± 1.0, *P* < 0.01) and None-SFBM (10.0 ± 2.0, *P* < 0.01) groups, followed by moderate lesions in the SF-only (8.5 ± 2.3, *P* < 0.01) group, and virtually no damage in the SFBM-both and Free plug groups (Fig. 3B; Table 2) at 12 weeks.

**Table 2:** Different group Mankin Score in 4, 8, and 12 weeks after the operation (M ± QR)

Group (weeks)	SFBM-both	BM-only	SF-only	None-SFBM	Free plug
4	1.0 ± 1.3	8.0 ± 1.0*^,▲^	1.0 ± 1.0	6.5 ± 3.0*^,▲^	0.5 ± 2.3
8	1.0 ± 2.0	9.5 ± 2.3*^,▲^	3.5 ± 1.8	8.5 ± 4.3*	1.0 ± 2.0
12	1.0 ± 2.0	14.0 ± 1.0*^,▲^	8.5 ± 2.3*	10.0 ± 2.0*	1.0 ± 1.5

* *P* < 0.005, vs. SFBM-both group; ^▲^ *P* < 0.005, vs. SF-only group

The thickness of cartilage samples from BM-only and None-SFBM groups was always significantly less than in the SFBM-both group (*P* < 0.01). The decrease was in the order of 60 % (0.6-fold) at 4 weeks, 27 % (3.7-fold) at 8 weeks, and 18 % (5.6-fold) at 12 weeks in the BM-only group (vs. SFMB-both) and 60 % (0.6-fold) at 4 weeks, 30 % (3.3-fold) at 8 weeks, and 21 % (4.8-fold) at 12 weeks in the None-SFBM group (vs. SFBM-both). The thickness of cartilage in the SF-only group was significantly lower than in the SFBM-both group at 8 and 12 weeks after operation (Fig. 3C; Table 3) (*P* < 0.01).

**Table 3:** Changes of cartilage thickness (μm) at 4, 8, and 12 weeks in different groups (mean ± SD)

Group (weeks)	SFBM-both	BM-only	SF-only	None-SFBM	Free plug
4	301.8 ± 29.5	180.9 ± 24.2**^,##^	290.6 ± 41.4	181.9 ± 19.9**^,##^	309.4 ± 18.3
8	309.2 ± 36.9	82.8 ± 30.6**^,##^	130.1 ± 27.1**	91.4 ± 13.23**^,##^	295.7 ± 22.2
12	300.6 ± 25.0	53.3 ± 12.8**^,##^	123.0 ± 16.6**	61.9 ± 23.5**^,##^	289.0 ± 18.9

* *P* < 0.05 vs. SFBM-both group, ** *P* < 0.01, vs. control group; ^#^ *P* < 0.05 vs. SF-only group, ^##^ *P* < 0.01, vs. SF-only group

Blood vessels, bone marrow, and channels were observed at the osteochondral junction of cartilage in BM-only groups at 4, 8, and 12 weeks after operation. We observed that the bone marrow started to invade into the calcified cartilage and subchondral bone, and blood vessels were crossing into or approaching cartilage at 4 and 8 weeks (Fig. 3D-a, b). Calcified cartilage disappeared and full thickness cartilage was lost at 12 weeks. In some areas, the residual cartilage was surrounded by fibril tissue and bone marrow (Fig. 3D-c).

### Immunohistochemistry (IHC) of type II collagen

**Fig. 4:**
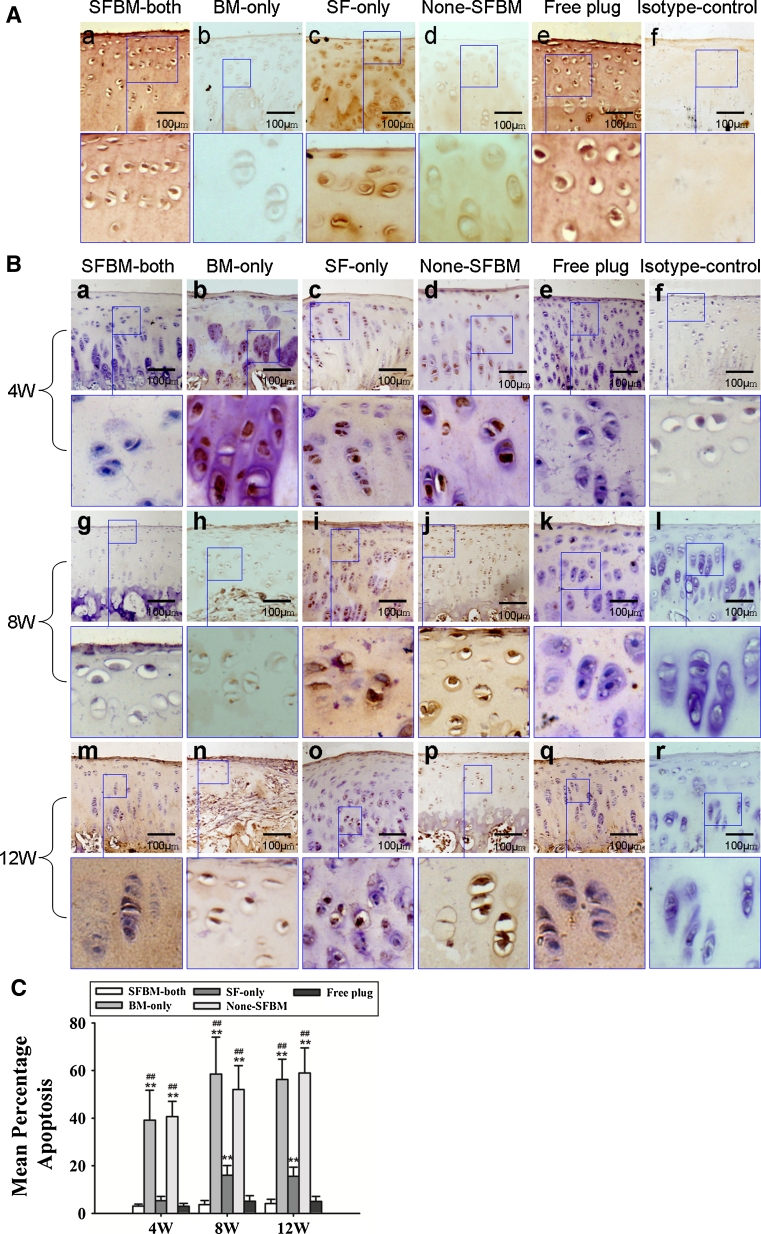
**A** Deprivation of nutrition resulted in decreased type II collagen staining at week 4. **B** The deprivation of nutrition resulted in an increase of chondrocyte apoptosis in the same group with time. **C** Percentage of apoptotic chondrocytes. * *P* < 0.05, vs. SFBM-both group, ** *P* < 0.01, vs SFBM-both group; # *P* < 0.05 vs. SF-only group, ## *P* < 0.01, vs. SF-only group

Strong type II collagen staining was observed in the SFBM-both and Free plug groups. However, type II collagen staining was significantly lower in the BM-only groups, followed by moderate increases in staining in the None-SFBM and SF-only groups. All BM-only, None-SFBM, and SF-only groups stained less for type II collagen than the SFBM-both and Free plug groups (Fig. 4A).

### Apoptosis of chondrocytes

The number of apoptotic chondrocytes in BM-only and None-SFBM groups was significantly higher than in SF-only, SFBM-both, or Free plug groups at all three time points. There were 39.1 % (*P* < 0.01), 58.5 % (*P* < 0.01), and 56.3 % (*P* < 0.01) more apoptotic cells at 4, 8, and 12 weeks, respectively, in cartilage from the BM-only group compared with cartilage from the SFBM-both or Free plug groups. And there were 40.6 % (*P* < 0.01), 52.1 % (*P* < 0.01), and 59.0 % (*P* < 0.01) more apoptotic cells at 4, 8, and 12 weeks, respectively, in cartilage from the None-SFBM group compared with cartilage from the SFBM-both or Free plug groups. The percentage of apoptotic chondrocytes in the SF-only group was 16 % (*P* < 0.01) and 15.6 % (*P* < 0.01) higher than in the SFBM-both groups at 8 and 12 weeks, respectively (Fig. 4C; Table 4).

**Table 4:** Percentage of apoptotic chondrocytes at 4, 8, and 12 weeks in different groups (mean ± SD)

Group (weeks)	SFBM-both	BM-only	SF-only	None-SFBM	Free plug
4	3.1 ± 0.8	39.1 ± 12.6**^,##^	5.3 ± 1.8	40.6 ± 6.4**^,##^	3.0 ± 1.2
8	3.7 ± 1.7	58.5 ± 15.5**^,##^	16.0 ± 4.1**	52.1 ± 10.0**^,##^	5.1 ± 2.3
12	4.2 ± 1.8	56.3 ± 8.5**^,##^	15.6 ± 3.9**	59.0 ± 10.5**^,##^	5.0 ± 2.2

* *P* < 0.05 vs. SFBM-both group, ** *P* < 0.01, vs. control group; ^#^ *P* < 0.05 vs. SF-only group, ^##^ *P* < 0.01, vs. SF-only group

### mRNA expressions of aggrecan, Type II Collagen, MMP-13

**Fig. 5:**
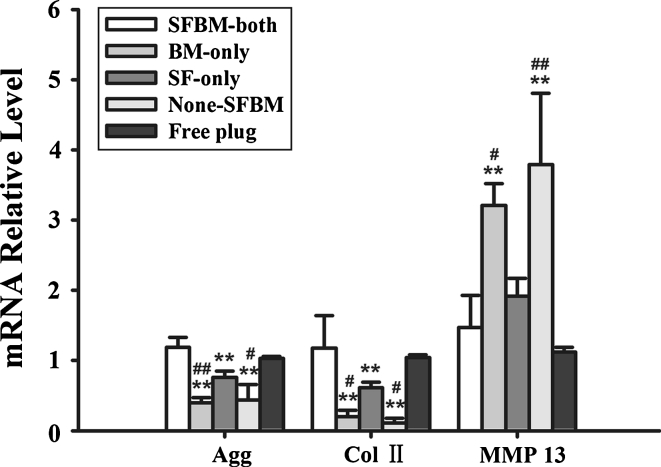
The deprivation of nutrition resulted in a decrease of Col II and Aggrecan mRNA, and an increase of MMP-13 mRNA expression at week 4 post surgery. * *P* < 0.05, vs. SFBM-both group, ** *P* < 0.01, vs SFBM-both group; # *P* < 0.05 vs. SF-only group, ## *P* < 0.01, vs. SF-only group

We observed that the aggrecan (AGG) and type II collagen (Col 2) mRNA levels in samples from the BM-only, None-SFBM, and SF-only groups taken at 4 weeks were significantly lower in comparison with the SFBM-both and Free plug groups: AGG and Col 2 contents were 40 % (*P* < 0.01) and 20 % (*P* < 0.01) lower in group BM-only; 44 % (*P* < 0.01) and 11 % (*P* < 0.01) lower in group None-SFBM; and 76 % (*P* < 0.01) and 61 % (*P* < 0.01) lower in group SF-only. However, the levels of MMP13 mRNA were significantly higher (3.2-fold in the BM-only group, *P* < 0.01, and 3.8-fold in the None-SFBM group, *P* < 0.01) compared with the SFBM-both group (Fig. 5; Table 5).

**Table 5:** Relative level of mRNA in different groups at 4 weeks (mean ± SD)

Group	SFBM-both	BM-only	SF-only	None-SFBM	Free plug
Agg	1.2 ± 0.1	0.4 ± 0.1**^,##^	0.8 ± 0.1**	0.4 ± 0.2**^,#^	1.0 ± 0.0
ColII	1.2 ± 0.5	0.2 ± 0.1**^,#^	0.6 ± 0.1**	0.1 ± 0.1**^,#^	1.0 ± 0.0
MMP13	1.5 ± 0.5	3.2 ± 0.3**^,#^	1.9 ± 0.3	3.8 ± 1.0**^,##^	1.1 ± 0.1

* *P* < 0.05 vs. SFBM-both group, ** *P* < 0.01, vs. control group; ^#^ *P* < 0.05 vs. SF-only group, ^##^ *P* < 0.01, vs. SF-only group

## Discussion

The most important finding of the present study was that SF-derived nutrition is the dominant source of sustenance for adult cartilage structure. When the only nutrition source is the BM, cartilage damage is observed due to blood vessel invasion from the bone marrow (Fig. 3D). Although extensive investigations have been performed to determine the sources of nutrition of articular cartilage, and the relationship between nutrition and cartilage degeneration [2, 6, 12, 17], the latter remains incompletely understood [15]. To determine which source of nutrition is more important in the maintenance of normal adult cartilage homeostasis, we designed a new PVC cap method to deprive cartilage from SF or BM nutrition (or both). Our results demonstrated that this simple method was effective at blocking cartilage’s nutrition from SF and BM. In addition, as demonstrated by the lack of detectable significant differences in the cartilage changes between the sham and SFBM-both control groups, the SFBM-both group could still obtain its nutrition from SF and BM, even if the cartilage plug was surrounded by a PVC tube.

It is well known that articular cartilage can get nutrition from both SF and BM [24]. However, which pathway is more important to articular cartilage homeostasis is still in argument [12, 17]. Our results proved that cartilage could be damaged by blockage of either nutrition pathway, but blocking the SF pathway resulted in significantly higher cartilage degeneration than blocking the subchondral BM pathway. Therefore, our findings suggest that articular cartilage is nourished mainly from the synovial fluid.

Malinin and Quellette [17] adopted bone cement walls to deprive nutrition of articular cartilage by subchondral bone, therefore focusing his studies on the impact of nutrition by subchondral bone. However, Malinin and Quellette’s model could not exclude heat damage due to the solidification of bone cement, and the occurrence of breaking after the solidification of bone cement may have influenced the effects of nutrition deprivation. Our study employed PVC implanted grafts, which possess friction characteristics close to articular cartilage, stable physicochemical qualities, and avoidable repulsive response [3, 13]. The implanted grafts in the control group showed good healing without any signs of inflammation or cartilage damage. Furthermore, the patellar cartilage did not show any signs of cartilage damage in BM-only group, which indicated that the implanted grafts in the femoral trochlear fit perfectly with its surrounded cartilage and did not induce any mechanical damage on the opposite patellar cartilage. Malinin and Quellette’s results indicated that interruption of contact between articular cartilage and vascularized subchondral bone resulted in degeneration of the cartilage, and detection of these degenerative changes required long time periods. Our results were similar to Malinin and Quellette’s results, but out experiment required a shorter time. Furthermore, we can compare the effects of simultaneous deprivation of nutrition from SF and SB. The presented evidence indicates that the PVC cap is a useful tool to investigate the effects of cartilage nutrition deprivation on cartilage degeneration.

The most severe cartilage damage was observed in the BM-only group, including BM blood invasion, calcified cartilage disappearance, cartilage damage, and surrounding of the residual cartilage by BM (Figs. 2A-q, 3A-b, g, D). Our data indicate that the SF may be the dominant source of nutrition necessary to maintain normal adult cartilage structure and function. Deficiency of nutrition by SF directly induces degeneration of cartilage. When nutrition from the SF was blocked, nutrition by BM became dominant. However, when BM became the main source of nutrition, it resulted in severe cartilage damage due to blood vessel invasion from the bone marrow. Studies have shown that the degeneration of cartilage is related with vascular invasion [7, 27, 28, 33]. Our result is consistent with these findings. Our findings indicate that modifying the nutrition of the synovial fluid or the prevention or blockage of blood invasion from the subchondral bone marrow may have a therapeutically effect in the treatment of cartilage degeneration.

Moderate cartilage damage was also found in the SF-only group, but this damage was less than in the BM-only group. This result indicates that nutrition from the bone morrow is also required for maintenance of normal cartilage homeostasis. It is interesting to notice that cartilage damage in the None-SFBM group did not cause further cartilage deterioration compared with BM alone. One possible explanation is the absence of blood vessel invasion (Figs. 2A-t, 3A-e, j).

PCR results further proved that blocking nutrition from either SF or BM resulted in decreased mRNA levels of AGG and Col II, which indicates that nutrition from both SF and BM are critical to maintain normal function of articular cartilage. MMP-13 plays a critical role in OA-related cartilage degeneration [29]. In this study, we noticed that MMP-13 mRNA was increased in the BM-only, None-SFBM, and SF-only groups compared with the SFBM-both and Free plug groups, which indicates that MMP-13 plays an important role in the cartilage degeneration caused by the deprivation of nutrition from SF or BM (Fig. 5).

The location for collection of cartilage samples is critical to cartilage thickness analysis, because the thickness of cartilage changes with anatomical location [5, 26]. In order to obtain consistent and comparable results, we chose the center of the femur trochlea, parallel to the upper edge of lateral ligament(Fig. 1A, B)in this study. The patellofemoral joint is an important weight loading joint in rodents. This joint is broad and flat above the upper edge of the lateral ligament. Cartilage from this site was homogeneous in thickness in the sagital plane of the femur trochlea. Therefore, the data obtained from this location were consistent and can be compared among the different groups.

## Conclusion

There are two routes for articular cartilage nutrition: diffusion from the synovial fluid or subchondral bone marrow. Nutrition from the synovial fluid is essential for normal cartilage structure and function, and the loss of this source of nutrition leads to much more severe cartilage degeneration compared with the loss of the nutrition from the subchondral bone marrow. Besides, nutrition from the subchondral bone marrow is also required for normal cartilage structure and function, and deterioration of cartilage may be caused by deficiency of nutrition from the subchondral bone marrow. Modifying the synovial fluid nutrition and preventing blood invasion from the subchondral bone marrow may have therapeutic effects in the cartilage degeneration.

## Electronic supplementary material

Below is the link to the electronic supplementary material.

Supplement 1: Instruments a: PVC cup; b: PVC tube; c: Trephine; d: 4 mm in diameter (TIFF 942 kb)

Supplement 2. A pilot experiment was carried out to test whether or not PVC cap can block the diffusion of nutrients into the cartilage plug. To test whether a PVC cap can block diffusion of nutrients into the cartilage plugs, a pilot experiment was carried out before the study. Two weeks after the model was created, 1 mL methylene blue was injected into the knee joints 1 day before the animals were sacrificed. a: No staining was found in the cartilage plug after 1 ml methylene blue was injected into the knee joints 1 day before the animals were sacrificed in the BM-only group (f). Two weeks post operation, the color of cartilage plugs in SF-only and None-SFBM groups turned pale (h, j) (white arrow) compared to its initial redness (c, e) (black arrow), demonstrating the definite efficacy of the PVC cap at deprivation of nutrition (TIFF 1721 kb)

Supplement 3. Measurement of cartilage thickness a: Measurement of cartilage thickness on the sagittal slices (y). b, c: Measurement of cartilage thickness by Imaga-Pro6.3 Software with microscopy (Olympus BX51; Olympus, Tokyo, Japan) (TIFF 570 kb)
