# Supplementary figures and images for: Nutrition and degeneration of articular cartilage
Source: Knee Surg Sports Traumatol Arthrosc. 2012 Apr 4;21(8):1751–62. doi: 10.1007/s00167-012-1977-7 (PMC3717153; doi:10.1007/s00167-012-1977-7)

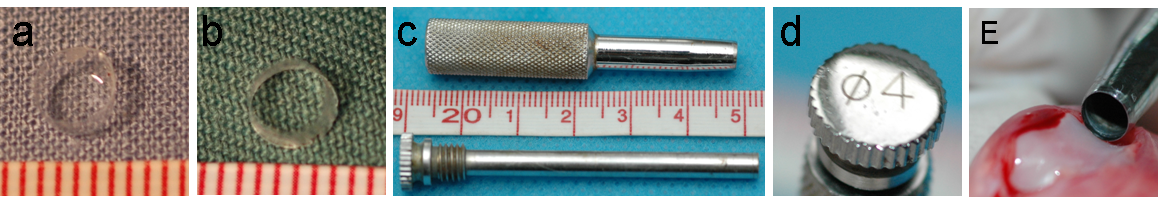

Supplement: Supplementary file 1 — Supplement 1: Instruments a: PVC cup; b: PVC tube; c: Trephine; d: 4 mm in diameter (TIFF 942 kb) [file 167_2012_1977_MOESM1_ESM.tif]

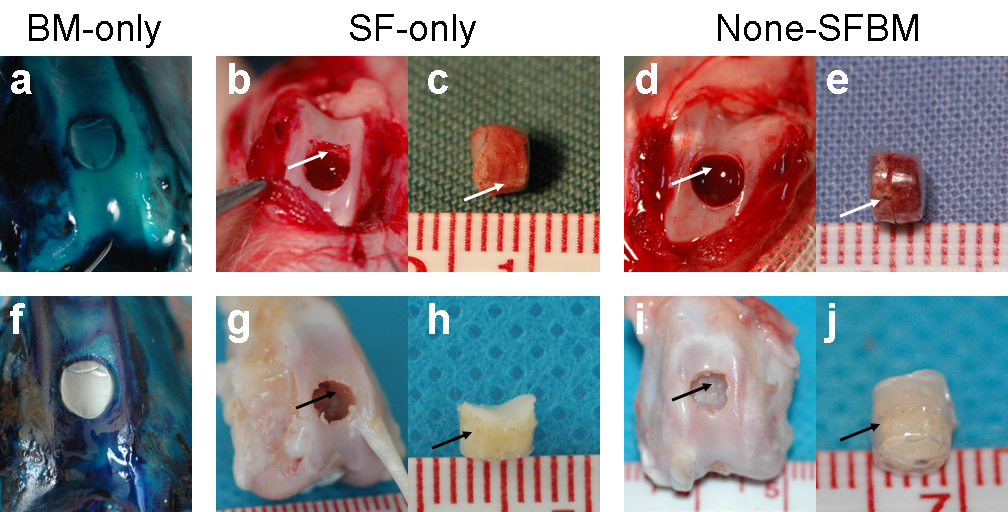

Supplement: Supplementary file 2 — Supplement 2. A pilot experiment was carried out to test whether or not PVC cap can block the diffusion of nutrients into the cartilage plug. To test whether a PVC cap can block diffusion of nutrients into the cartilage plugs, a pilot experiment was carried out before the study. Two weeks after the model was created, 1 mL methylene blue was injected into the knee joints 1 day before the animals were sacrificed. a: No staining was found in the cartilage plug after 1 ml methylene blue was injected into the knee joints 1 day before the animals were sacrificed in the BM-only group (f). Two weeks post operation, the color of cartilage plugs in SF-only and None-SFBM groups turned pale (h, j) (white arrow) compared to its initial redness (c, e) (black arrow), demonstrating the definite efficacy of the PVC cap at deprivation of nutrition (TIFF 1721 kb) [file 167_2012_1977_MOESM2_ESM.tif]

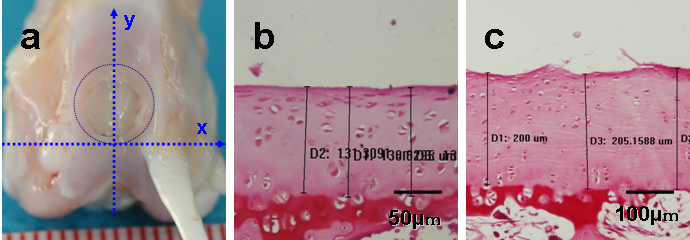

Supplement: Supplementary file 3 — Supplement 3. Measurement of cartilage thickness a: Measurement of cartilage thickness on the sagittal slices (y). b, c: Measurement of cartilage thickness by Imaga-Pro6.3 Software with microscopy (Olympus BX51; Olympus, Tokyo, Japan) (TIFF 570 kb) [file 167_2012_1977_MOESM3_ESM.tif]
